# PyMod: sequence similarity searches, multiple sequence-structure alignments, and homology modeling within PyMOL

**DOI:** 10.1186/1471-2105-13-S4-S2

**Published:** 2012-03-28

**Authors:** Emanuele Bramucci, Alessandro Paiardini, Francesco Bossa, Stefano Pascarella

**Affiliations:** 1 Dipartimento di Scienze Biochimiche "A. Rossi Fanelli", Sapienza Università di Roma, Roma 00185, Italy

## Abstract

**Background:**

In recent years, an exponential growing number of tools for protein sequence analysis, editing and modeling tasks have been put at the disposal of the scientific community. Despite the vast majority of these tools have been released as open source software, their deep learning curves often discourages even the most experienced users.

**Results:**

A simple and intuitive interface, PyMod, between the popular molecular graphics system PyMOL and several other tools (i.e., [PSI-]BLAST, ClustalW, MUSCLE, CEalign and MODELLER) has been developed, to show how the integration of the individual steps required for homology modeling and sequence/structure analysis within the PyMOL framework can hugely simplify these tasks. Sequence similarity searches, multiple sequence and structural alignments generation and editing, and even the possibility to merge sequence and structure alignments have been implemented in PyMod, with the aim of creating a simple, yet powerful tool for sequence and structure analysis and building of homology models.

**Conclusions:**

PyMod represents a new tool for the analysis and the manipulation of protein sequences and structures. The ease of use, integration with many sequence retrieving and alignment tools and PyMOL, one of the most used molecular visualization system, are the key features of this tool.

Source code, installation instructions, video tutorials and a user's guide are freely available at the URL http://schubert.bio.uniroma1.it/pymod/index.html

## Background

Once confined only to experts in bioinformatics, protein sequence retrieving, aligning and modeling tasks are now being routinely approached by an increasing number of researchers, who can take also advantage of the growing number of structures that are being deposited every day in public databases. Integrating protein sequence and structure information has therefore become an imperative, especially in the field of protein structure prediction from sequence, by means of homology modeling (HM) methodologies.

In recent years, a number of valuable tools related to protein sequence analysis and modeling (e.g., DeepView [1], MolIDE [2] and Chimera [3]) has been developed. While these tools are in many cases easily accessible, and have greatly simplified some of the problems that are most frequently encountered when coping with sequence/structure analysis tasks (e.g., lack of graphical user interfaces [GUIs], need to make use of many programs in an integrated way and input and output file format manipulation problems), the initial difficulties and deep learning curves often encountered when mastering the usage of new software sometimes discourages first-time, as well as more experienced users. On the other hand, public servers (e.g., Phyre [4], CPHmodels [5]), which are able to automatize some or all of the main modeling tasks, often do not offer users the ability to apply knowledge-based intervention during the analysis (e.g., sequences selection, manual refinement of multiple alignments and choice of parameters during model construction).

**Figure 1:**
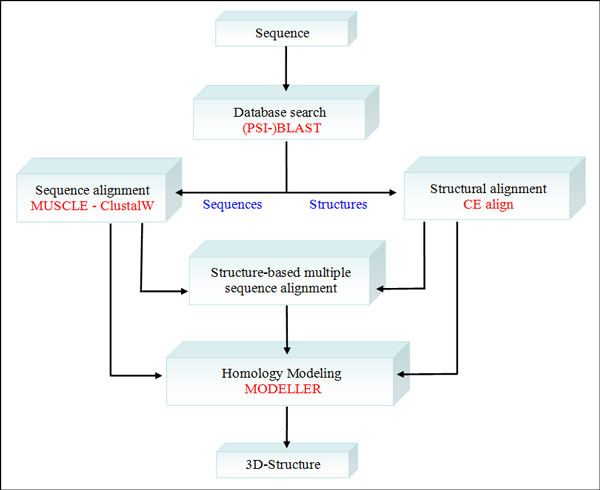
**PyMod integrated tools flowchart**. The workflow shows how the separate tools are integrated in PyMod. Each tool must be considered as *standalone *(e.g., it's possible to perform a sequence alignment task without searching in the database as a mandatory step).

In order to contribute to tackle these issues, a simple and intuitive interface between the open-source and widely used biomolecular visualization program PyMOL [6] and several other well-known sequence/structure analysis tools (i.e., BLAST [7], PSI-BLAST [8], MUSCLE [9] ClustalW [10], CEalign [11] and MODELLER [12]; Table 1), has been developed. The tool presented here, PyMod, aims to give researchers and students with no or a limited familiarity in this field, as well as more experienced users, the ability to exploit popular algorithms in sequence/structure analysis and protein structure prediction, and most importantly full customization and control over their parameters, while retaining as much as possible an ease of use and the familiarity of the PyMOL environment (Figure 1).

**Table 1:** PyMod integrated tools

**BLAST**	http://blast.ncbi.nlm.nih.gov/Blast.cgi
**PSI-BLAST**	http://www.ncbi.nlm.nih.gov/blast/Blast.cgi?CMD=Web&PAGE=Proteins&PROGRAM=blastp&RUN_PSIBLAST=on
**MUSCLE**	http://www.drive5.com/muscle/
**ClustalW**	http://www.ebi.ac.uk/Tools/msa/clustalw2/
**Cealign**	http://cl.sdsc.edu/ce.html
**Modeller**	http://salilab.org/modeller/

This table summarizes the tools that have been integrated into PyMod providing their URLs.

## Implementation

PyMod has a rich functionality, based on its core sequence alignment, clustering and editing window. These features are described in outline in the following sub-sections.

### Similarity searches

**Figure 2:**
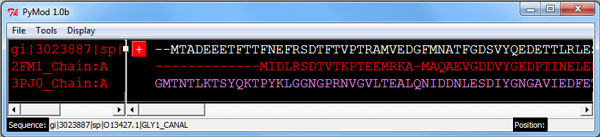
**PyMod main window**. By using the main window, the user can perform almost all PyMod functions using the top menu or just operating on the sequences and their name.

**Figure 3:**
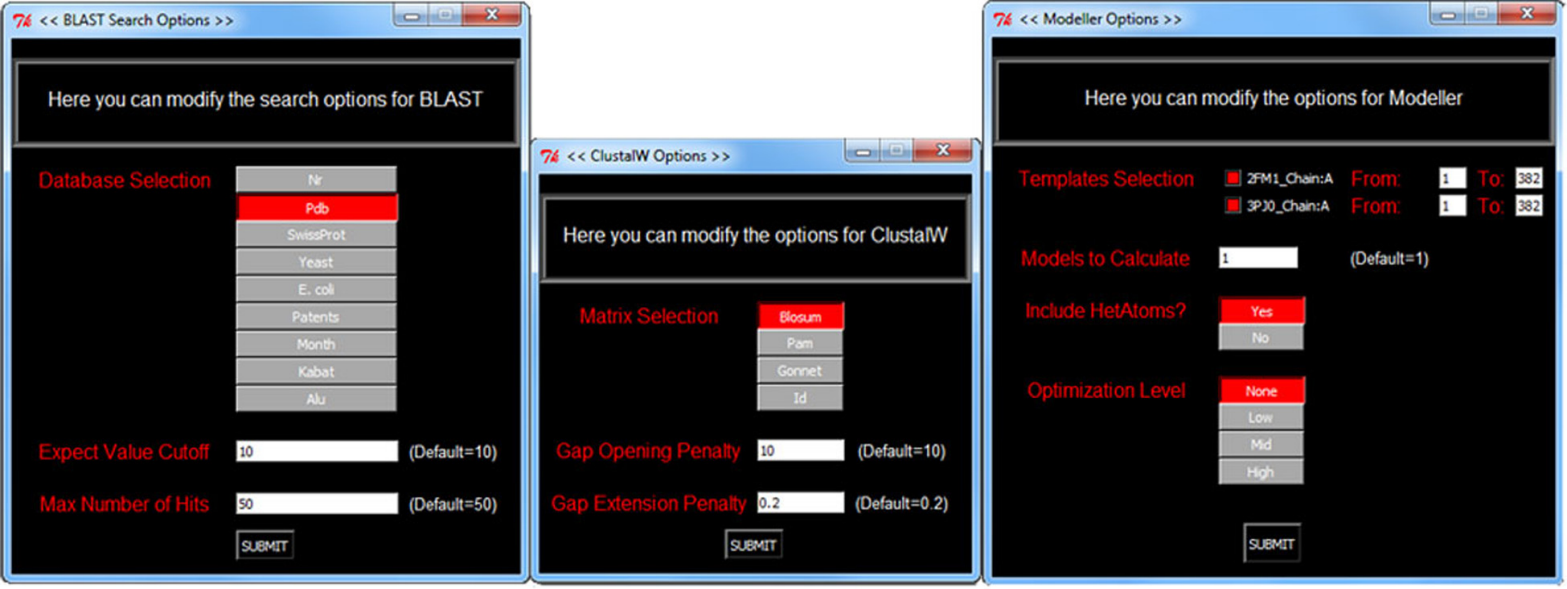
**Preferences window**. The user can change many parameters of the tools implemented in PyMod through specific Options windows.

PyMod can input and output sequences and 3D-structures in the popular FASTA and PDB formats. In the latter case, 3D-coordinates are automatically split in single chains, loaded into PyMOL, and their corresponding sequences loaded into the PyMod main window (Figure 2). After a sequence has been loaded onto the PyMod main window, users can search different databases, in order to retrieve protein sequences and related structures that are homologous to the query sequence, by means of the BLAST and PSI-BLAST search tools. BLAST is relatively faster while less sensitive when compared with profile-profile alignment methods. However, it can still detect homology with significant sequence identity (i.e., identity > 40%) [8,13,14], thus providing fast and useful means in the case of high identity, template-based modeling. On the other hand, PSI-BLAST, the most used profile-sequence alignment method, is more sensitive than sequence-sequence alignment and it can recognize distant homology with lower sequence identity (i.e., identity > 20%) [8]. Both tools have been therefore implemented in PyMod. Profile-profile alignments or HMM-HMM (Hidden Markov Models) comparison algorithms [15] may be the most effective approaches and even able to create accurate alignments in extreme cases (i.e., identity < 10%) [16], but they're usually much more complex and slower than sequence-sequence or profile-sequence alignments. Most notably, at these levels of sequence identity (0-20%), fold-recognition or *ab initio *approaches may be favored over homology modeling, for which PyMod flowchart has been primarily planned. PyMod includes support for running BLAST remotely (no local database installation is required) and PSI-BLAST locally. In the latter case, users are provided with the option to install local sequence databases, while PyMod provides a graphical interface to ease their use. To facilitate template structure search for homology modeling tasks, PyMod will be distributed with a pre-installed PDB sequence database, which will be updated in future releases on a monthly base. A number of (PSI-)BLAST parameters can be controlled by the user from within an apposite PyMod window (e.g., number of PSI-BLAST iterations, E-value threshold, % identity threshold) (Figure 3). Users are provided with the ability to select the (PSI-)BLAST results to be imported in the PyMod main window, by choosing from a table reporting the name of the retrieved sequences, their E-value and sequence identity. Selected sequences, once imported in PyMod, are automatically grouped in a separate cluster, which can be collapsed or contracted by simply clicking a button beside the query sequence. When searching the PDB database the user can retrieve the 3D-coordinates that are related to a selected query and automatically load the structure into the PyMOL main window.

As such, PyMod provides a graphical interface for (PSI-)BLAST searches of large databases, both locally or remotely, which can be also used as a standalone tool inside the PyMOL framework.

### Alignment of sequences and structures

**Figure 4:**
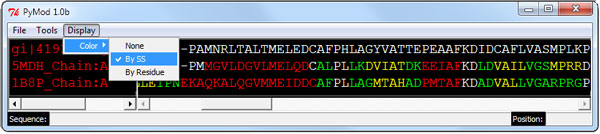
**Secondary structure colouring method**. The user can color the protein sequences by their secondary structure or by the physicochemical properties of their amino acids. The secondary structure assignment is based on the proSS algorithm http://roselab.jhu.edu/utils/pross.html.

Once retrieved sequences from selected databases are loaded in PyMod, they can be used to generate a multiple sequence alignment by means of MUSCLE and ClustalW programs. The choice of two multiple sequence alignment tools is twofold: on the one hand, ClustalW is famous and very popular among people with limited experience in the field; on the other hand, MUSCLE is known to outperform ClustalW in quality and in speed. Future implementation of additional tools (e.g., T-Coffe [17]) is planned. Additionally, PyMod can input and output multiple alignments in the popular FASTA and Clustal formats. Different multiple alignments can be built for each cluster of sequences that is available in PyMod. The user from within an apposite PyMod window can control a number of ClustalW parameters. When dealing with sequence alignments comprising known 3D-structures, it is always more desirable to exploit this kind of information, by performing structural superposition and deriving a structure-based alignment. In this case, users can carry out a multiple structural alignment by using the combinatorial extension algorithm, implemented in the popular program CE, a fast and robust algorithm in superposing and aligning 3D-structures [11]. The selected 3D-structures are then automatically superposed in PyMOL, and the resulting structural alignment is displayed in PyMod. If the 3D-structures to be superposed and aligned have been previously aligned to their own sequence cluster with MUSCLE or ClustalW, users can optionally keep the latter, by using the structural alignment as guide to "merge" the two alignments. In this way, structure-based alignments are used as a template for realigning the original sequences, obtaining a structure-based multiple sequence alignment that combines sequences and structures. This procedure is similar to the one already implemented in the 3DCoffe tool [18]. The option to generate mixed structure-sequence alignment is particularly useful when two or more evolutionarily distant structural templates and their close orthologous sequences have to be aligned. In this scenario, the structural alignment of templates (which, being based on structure superposition, would outperform any sequence-based method) will provide the starting point for the subsequent merging of orthologous sequences, which have been previously aligned with MUSCLE or ClustalW. Most importantly, manually editing multiple sequence alignments in PyMod will allow the user to apply her/his knowledge to correct any misaligned residue. This option in PyMod simply requires the user to click with the mouse at the desired sequence position and then to drag residues to the right/left to add/remove gaps. The ability to edit sequences is another feature implemented in PyMod. Indeed, during modeling tasks, it is often necessary to mutate and/or trim existing sequences at their ends. This option, for example, helps to prevent long overhanging fragments after a MODELLER run. Excising part of the sequence in the middle is also possible. Finally, a number of coloring options for sequences are available via the PyMod menu, including a secondary structure scheme for sequences related to 3D-structures (Figure 4).

### Homology modeling

**Figure 5:**
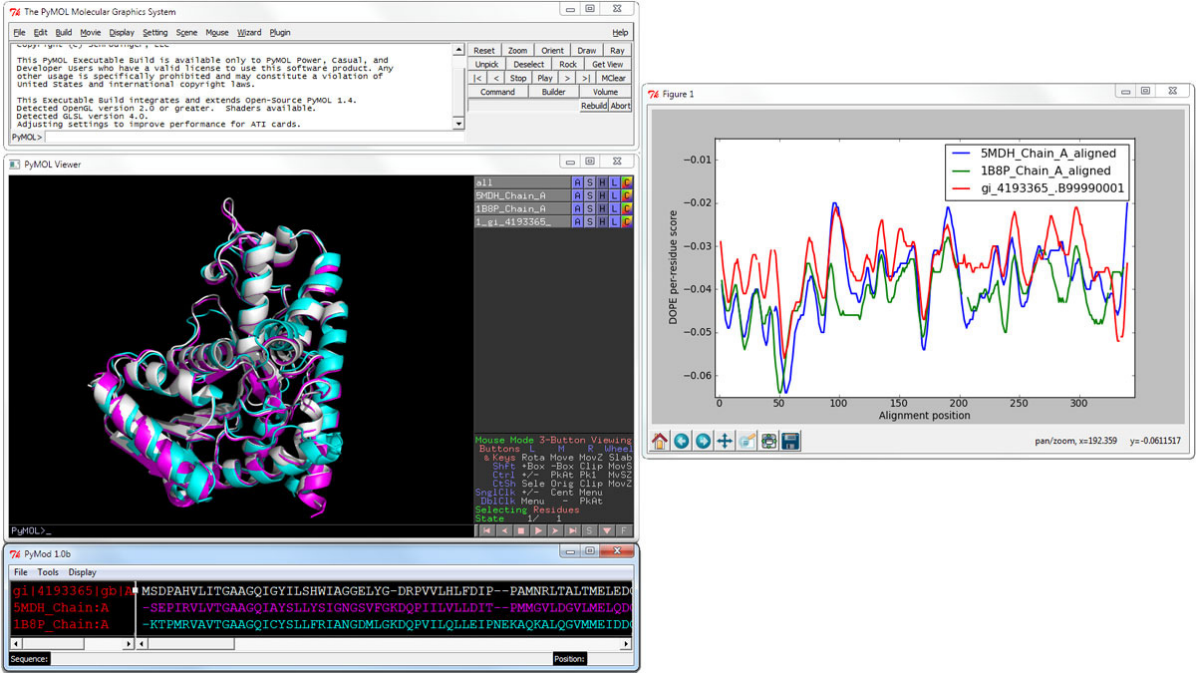
**Homology model and quality assessment**. Once homology models have been built, they are automatically loaded onto PyMOL and the user can assess the models quality through a graph constructed using the DOPE scoring function.

Starting from a previously obtained alignment, it is possible to build a homology model of a selected sequence through the PyMod interface to the popular MODELLER program. The satisfaction of spatial restraints algorithm, as implemented in MODELLER, undoubtedly represent one of the most popular homology modelling approaches, and has become the model-building program of choice for several homology modelling servers because of its relative speed and reliability. Several of the strongest performing prediction servers in the CASP8 experiment, such as HHpred [19], incorporate MODELLER in their methodology. When compared against other homology modeling programs MODELLER is considered one of the better performing structure predictors [20]. Up till now, there have been a few attempts earlier to simplify the use of MODELLER by providing a GUI framework (EasyModeller [21], SWIFT MODELLER [22]). Merging MODELLER with the most popular tools for sequence retrieving and sequence/structure alignments ([PSI-]BLAST, ClustalW, MUSCLE, CE), within the PyMOL framework gives an unprecedented level of ease and control over all of the tasks required to construct homology models with MODELLER. A number of MODELLER parameters can be controlled by the user from within an apposite PyMod window. These include the choice of structural template(s), the model refinement level, the number of models to build, and the specification of the region to be modeled. By merging the versatility of MODELLER and the user-friendly PyMod/PyMOL environment, it is also possible to easily include hetero-atoms (e.g., inhibitors, docked substrates, cofactors) in the final models, a pivotal feature that is often absent in many state of the art tools for homology modeling. Finally, the pipeline includes a validation tool (DOPE, or Discrete Optimized Protein Energy [23]), to highlight regions where the alignment/model is accurate and where it is likely to be incorrect (Figure 5). Once homology models have been built, they are automatically loaded onto PyMOL for visual inspection and further analysis.

## Conclusions

PyMod represents a new tool for the analysis and the manipulation of protein sequences and structures. The ease of use, integration with many sequence retrieving and alignment tools and PyMOL, one of the most used molecular visualization system, are the key features of this tool. We plan to release future updates of PyMod, including additional tools for secondary structure prediction, sequence retrieving and alignment, as well as other tools suggested by the users' community. Finally, a tighter integration between PyMOL, MODELLER and PyMod will constitute a main issue of future project development plans.

## Availability and requirements

Project name: PyMod

Project home page: http://schubert.bio.uniroma1.it/pymod

Operating system(s): Windows (XP, Vista, Seven). Linux (Ubuntu) and Mac OS (10.6) will be supported in the next release.

Programming language: Python

License: Lesser General Public License (LGPL)

Other requirements: PyMOL version 1.1.1 or newer, BioPython version 1.50 or newer, Standalone BLAST 2.2.25+ or newer, Muscle, ClustalW and MODELLER.

## Competing interests

The authors declare that they have no competing interests.

## Authors' contributions

EB wrote the software and helped to draft the manuscript. AP conceived the study, helped to write the program and drafted the manuscript. FB revised the manuscript critically for important intellectual content. SP participated in the study design and coordination and revised the manuscript. All authors read and approved the final manuscript. EB and AP contributed equally to this work.
